# Narrative Review of Artificial Intelligence in Ophthalmic Disease Detection: Artificial Intelligence in Ophthalmic Diseases Detection

**DOI:** 10.31661/gmj.v14i.3979

**Published:** 2025-09-20

**Authors:** Kholoud Ahmad Bokhary

**Affiliations:** ^1^ Department of Optometry, College of Applied Medical Science, King Saud University, Riyadh, Saudi Arabia

**Keywords:** Artificial Intelligence, Machine Learning, Ophthalmology, Diagnostic Imaging, Eye Diseases

## Abstract

**Background:**

Artificial intelligence (AI) is revolutionizing ophthalmology and optometry by utilizing high-resolution imaging modalities such as optical coherence tomography (OCT), fundus photography, and corneal topography. These modalities generate quantifiable data suitable for machine learning (ML), facilitating automated diagnosis of ocular conditions like diabetic retinopathy, glaucoma, and age-related macular degeneration (AMD), which are leading causes of visual impairment worldwide. This narrative review evaluates the role of ML in improving diagnostic accuracy and accessibility in eye care, focusing on methodological complexities, supervised and unsupervised learning approaches, and challenges in clinical integration.

**Materials and Methods:**

A comprehensive narrative literature review was conducted, analyzing ML applications in ophthalmology.

**Results:**

AI systems exhibit high sensitivity and specificity, often outperforming human graders in diabetic retinopathy screening and early detection of glaucoma and AMD using OCT and fundus imaging. Anterior segment diseases benefit from AI-driven corneal topography analysis. Challenges include image quality, dataset imbalances, and variability in imaging protocols, necessitating fine-tuning for diverse clinical environments. Unsupervised learning shows potential for identifying novel biomarkers but requires further validation.

**Conclusion:**

AI-driven ML models significantly enhance eye disease diagnostics, improving accuracy and accessibility, particularly in resource-limited settings. However, challenges like data standardization and model generalizability must be addressed to ensure robust clinical adoption.

## Introduction

Artificial intelligence (AI) is changing the future of medicine by improving diagnostic accuracy and streamlining patient care [1]. As a visually reliant specialty, both ophthalmology and optometry have been at the forefront of the implementation of AI due to the fact that it relies on high-resolution imaging modalities such as optical coherence tomograph, fundoscopy, and corneal topography [2][3]. These imaging modalities provide standardized data that can be quantified, which makes them ideal for machine and deep learning algorithms [2][3]. Ocular conditions such as diabetic retinopathy, glaucoma, and age-related macular degeneration are among the leading causes of visual disability worldwide [4][5][6]. Early detection, diagnosis and treatment are important to mitigate permanent loss of vision. AI systems have shown high potential for automating the diagnosis and grading of these conditions, thereby optimizing diagnostic efficiency, reducing clinician workload, as well as enhancing access to treatment, particularly in under-staffed resource-limited settings where specialist care may not be readily available [7] and even as a treatment modality [8]. Diabetic retinopathy, the most studied condition for AI usage in this era, benefits significantly from AI-driven screening using fundus photography, with studies like Huang et al. (2022) [9] and Grzybowski et al. (2020) [10] demonstrating high sensitivity and specificity, often surpassing human graders in primary care and low-resource settings, enhancing accessibility and reducing vision loss [7]. However, challenges such as image quality, patient cooperation, and integration into diverse healthcare systems persist, requiring fine-tuning for heterogeneous clinical environments. In glaucoma, AI excels in detecting early optic nerve damage and predicting visual field loss through optical coherence tomography (OCT) and visual field testing, as shown by Li F et al. (2024) [11], offering precise segmentation and metrics for clinical decision-making, though variability in imaging protocols limits generalizability. For AMD, AI models, as explored by Wei W et al. (2023) [12], accurately identify and grade lesions like geographic atrophy and drusen using fundus and OCT, supporting early intervention and progression forecasting, yet face issues with dataset imbalance and model interpretability. Anterior segment diseases, including keratoconus, cataracts, and angle-closure glaucoma, benefit from AI’s ability to analyze corneal topography and anterior segment OCT for early diagnosis, severity grading, and surgical planning, as noted in studies by Nguyen T et al. (2024) [13], Soh ZD et al. (2024) [14], and Wu X et al. (2020) [15], though variability in imaging devices and diagnostic standards poses challenges. In this narrative review, we are going to read literature focused on methodological complexities of ML for eye diseases, to provide an introduction for clinicians to get familiar with fundaments of ML in medical imaging.

## Fundamentals of Machine Learning for Eye Care

**Table:** Table1. Master Summary of Included Studies

**Dataset**	**Source**	**Imaging Modality **	**Size**	**Diseases Covered **	**Key Features**	**Limitations**
OCTDL	Kulyabin et al. (2024) [29]	OCT	2,000+ images	AMD, DME, ERM, RAO, RVO, VID	Labeled by retinal specialists, high-resolution, open-access	Limited representation of rare conditions
Duwairi et al.	Duwairi et al. (2021) [30]	OCT	21,991 images	CNV, Full/Partial Macular Hole, CSR, Geographic Atrophy, MRO, VMT	Annotated by Jordanian ophthalmologists, binary (84.90%) and multi-class (63.68%) classification	Lower multi-class accuracy, data imbalance
PAPILA	Kovalyk et al. (2022) [31]	Fundus	Not specified	Glaucoma	Includes both eyes, optic disc/cup segmentation, ResNet-50 tested	Limited to glaucoma, dataset size not detailed
RFMiD 2.0	Panchal et al. (2023) [32]	Fundus	860 images	AMD, DR, cataracts, glaucoma, rare diseases	Multi-class, multi-label, annotated by three eye specialists	Small dataset size, regional focus (Maharashtra)
FIVES	Jin et al. (2022) [33]	Fundus	800 images	Vessel segmentation for multiple conditions	High-resolution, pixel-wise annotations, crowdsourced by experts	Limited to vessel segmentation, scarce for other tasks

**OCTDL:** Optical Coherence Tomography Dataset for Image-Based Deep Learning; **RFMiD:** Retinal Fundus Multi-Disease Image Dataset; **FIVES:** fundus image vessel segmentation;

Machine learning (ML) models’ primary core stone is simply to train models based on images. This process involves multiple steps with wide range of methodological details that result in models that are able to classify images. AI models that could analysis images are known widely as vision models. Currently many efforts has been done in ophthalmology in this case and various datasets of eye images are established for detecting and diagnosing eye diseases, primarily through retinal imaging modalities like optical coherence tomography (OCT) and fundus photography. Image annotation plays a crucial role in training machine learning models for diagnosing eye diseases, with several datasets and methodologies demonstrating its impact. There are multiple studies, published datasets, pre-trained models on repositories of AI models like tensorflow like the EyeHealer dataset that provides large-scale, pixel-level annotations of anterior eye segment structures and lesions, enabling improved segmentation performance in deep learning models for anterior segment diseases [16]. Similarly, Li et al. highlighted that dense anatomical annotations of slit-lamp images enhance diagnostic accuracy by training models with both structural and pathological labels [17]. Crowdsourcing has also been explored as a cost-effective alternative to expert annotation, with studies showing that non-expert annotations of retinal images can achieve high agreement with expert assessments when proper training and consensus thresholds are applied [18]. Additionally, Camilo et al. introduced a comprehensive pupillary image dataset with manual annotations for glaucoma, diabetes, and alcohol-related conditions, facilitating the development of robust segmentation algorithms [19]. For OCT imaging, OCT5k offers multi-disease, multi-graded annotations to support automated retinal layer segmentation, while Soul leverages a human-machine collaborative framework to generate high-quality annotations for branch retinal vein occlusion (BRVO) cases [20][21]. Guidelines for glaucoma imaging annotation further standardize the process, ensuring consistency in labeling optic disc, retinal nerve fiber layer, and anterior chamber structures for AI applications [22]. Moreover, Punithavathi et al. demonstrated the effectiveness of SVM with active learning in automating retinal image annotation, achieving high precision in disease classification [23].

At the core of the methodological aspects of machine deep learning for the eye disease detection is Python, a versatile programming language that acts like a digital toolbox, allowing developers to write scripts that handle everything from image manipulation to complex calculations without needing advanced coding expertise upfront [24]. Common packages like PyTorch and TensorFlow serve as ready-made frameworks for building neural networks, think of them as pre-assembled engines that power machine learning models to "learn" patterns in images, such as identifying irregular shapes in the cornea or retina. For image preprocessing, libraries like OpenCV function as image editors on steroids, enabling simple tasks like converting colorful eye scans to grayscale for clearer focus or enhancing contrast to highlight abnormalities, while tools like Keras simplify the creation of convolutional neural networks, which are specialized algorithms that scan images layer by layer to detect features like blood vessels or ulcers [24].

The SUSTech-SYSU dataset, comprising 712 fluorescein-stained ocular images, facilitates advanced segmentation and classification of corneal ulcers, addressing the scarcity of high-quality datasets for supervised learning in ophthalmology [25]. This dataset includes detailed annotations for flaky corneal ulcers and three-tiered classification labels, general ulcer patterns, specific types, and severity grades. The baseline methodology employs adjacent scale fusion and corneal position embedding within a convolutional neural network, leveraging Python and PyTorch for training on an RTX 3090 GPU with CUDA 11.0 [26].

Retinal vessel segmentation and eye disease classification have also advanced through deep learning frameworks, notably fully convolutional neural networks (FCNs) and U-Net models. One approach integrates stationary wavelet transform for multiscale analysis with an FCN, using rotation-based data augmentation and prediction refinement to achieve high sensitivity (0.8315) and specificity (0.9858) on datasets like DRIVE, STARE, and CHASE_DB1 [27]. Another project employs CNNs inspired by VGG-16 for multi-label classification of retinal abnormalities, such as diabetic retinopathy and glaucoma, with preprocessing steps like grayscale conversion and Keras ImageGenerator augmentation to address class imbalances, achieving a validation accuracy of 92% [25]. Ensemble methods, such as stacking InceptionV3, VGG19, and InceptionResNetV2 into a meta-neural network, showed a 98.31% accuracy for cataract detection, demonstrating robustness through high precision and sensitivity [25].

Challenges like overfitting and limited dataset size persist, particularly in diabetic retinopathy classification, where a deep convolutional neural network with white top-hat preprocessing and binary cross-entropy loss achieves a test accuracy of 63% on the IDRiD dataset [25]. The HEI-MED dataset, with 169 fundus images, supports exudate-based diabetic macular edema detection, utilizing manual segmentations and automated vasculature analysis to enhance diagnostic precision [28].

Other notable datasets include OCTDL, which contains over 2,000 OCT images labeled for diseases such as age-related macular degeneration (AMD), diabetic macular edema (DME), epiretinal membrane (ERM), retinal artery occlusion (RAO), retinal vein occlusion (RVO), and vitreomacular interface disease (VID), acquired using an Optovue Avanti RTVue XR and annotated by retinal specialists for deep learning applications [29]. Another significant dataset, provided by Duwairi et al., comprises 21,991 OCT images from Jordan, covering seven eye diseases (e.g., choroidal neovascularization, macular holes, central serous retinopathy) and normal cases, achieving 84.90% accuracy in binary classification and 63.68% in multi-class classification using a modified U-Net model [30]. The PAPILA dataset focuses on glaucoma, offering fundus images and clinical data from both eyes of patients, annotated for optic disc and cup segmentation, and tested with ResNet-50 for classification [31]. The RFMiD 2.0 dataset, with 860 fundus images annotated for multiple diseases including AMD, diabetic retinopathy, and rare conditions, supports multi-class and multi-label classification, collected from patients in Maharashtra, India [32]. Lastly, the FIVES dataset provides 800 high-resolution fundus images with pixel-wise vessel segmentation, aimed at enhancing AI-based vessel analysis for various clinical conditions [33]. These datasets, while advancing AI-driven diagnostics, face challenges such as data imbalance, limited annotations for rare diseases, and variability in imaging protocols, necessitating further standardization and validation for robust clinical application.

## Supervised Learning and Deep Learning Models for Disease Classification

Supervised learning is a fundamental paradigm in machine learning where models are trained on labeled datasets to predict outcomes or classify data based on input features. In this approach, the algorithm learns from examples that include both input data and corresponding correct outputs, enabling it to map relationships between them. During training, the model adjusts its parameters to minimize errors between predicted and actual labels, often using techniques such as backpropagation in neural networks [34][35]. This method contrasts with unsupervised learning, which deals with unlabeled data, and is particularly effective in tasks requiring high accuracy, such as medical image classification, where precise annotations guide the learning process [34][35].

Recent advancements in supervised learning have significantly enhanced the classification of eye diseases using retinal imaging, with convolutional neural networks (CNNs) emerging as a dominant architecture. Models like ResNet50 and DenseNet121 have been employed to classify conditions such as cataracts, diabetic retinopathy, and glaucoma from color fundus photography, achieving high accuracy rates on diverse datasets [36]. These supervised approaches leverage pre-trained networks to extract hierarchical features from images, enabling effective differentiation between normal and pathological states. In another study, deep learning models including VGGNet and MobileNet were trained on large retinal scan datasets to predict multiple ocular disorders, outperforming traditional machine learning methods like support vector machines with accuracies exceeding 98% [37]. Such supervised frameworks benefit from explicit label guidance, which improves model generalization in clinical settings, though they often require substantial annotated data to mitigate overfitting. Supervised learning remains a cornerstone for accurate eye disease diagnosis, with ongoing research focusing on optimizing architectures to reduce dependency on large labeled datasets while preserving clinical utility [36][37][38][39][40][41][42].

Supervised learning techniques have also been integrated into multi-modal frameworks for improved eye disease detection, combining retinal images with other data sources to boost diagnostic precision. A novel architecture fusing fundus images, optical coherence tomography scans, and clinical metadata through CNN-based feature extraction demonstrated superior performance in identifying cataracts and glaucoma, with accuracy rates reaching 95% [42]. This supervised fusion strategy enhances feature representation by learning from labeled multi-source inputs, addressing challenges like image variability. Similarly, supervised models applied to out-of-distribution datasets have shown robustness in glaucoma classification, using normalized loss functions to handle data shifts and maintain high AUC scores [41].

Despite their strengths, supervised learning models for eye disease classification face limitations in data-scarce environments, prompting explorations of hybrid approaches. For example, CNN architectures trained under supervised paradigms on datasets like EyeQ and AIROGS achieved promising results in multi-class glaucoma grading, with sensitivities around 93% [39]. However, comparisons with self-supervised alternatives highlight that while supervised methods excel with ample labels, they may underperform when annotations are limited, as seen in OCT image classification tasks where supervised baselines lagged behind multi-stage models [40].

Advancements in deep learning have significantly improved the automated classification of eye diseases through the analysis of retinal and Optical Coherence Tomography (OCT) scans.

Researchers have employed CNNs to create robust systems for identifying multiple ocular conditions, with a particular emphasis on myopia while extending capabilities to disorders like diabetic retinopathy, glaucoma, cataract, and age-related macular degeneration. Through the use of transfer learning and model fine-tuning, these approaches have attained impressive accuracy levels, such as over 97% in myopia detection, showing the potential of AI to enhance diagnostic precision and facilitate early intervention in vision care [43].

Comparative assessments of deep learning frameworks, encompassing CNNs, Transformer-based models, and efficient lightweight variants, reveal their strengths in multi-class ocular disease identification. Techniques like data augmentation and transfer learning have been instrumental in overcoming dataset imbalances, leading to superior outcomes in detecting intricate pathologies such as diabetic retinopathy. These insights offer practical recommendations for designing AI tools that balance accuracy with computational demands, ultimately aiding the development of effective clinical aids [44].

Innovative applications of architectures like EfficientNetB3 have demonstrated strong performance in classifying eye ailments from fundus imagery, achieving around 93% overall accuracy across categories including cataract, glaucoma, diabetic retinopathy, and normal states. Complementing this, hybrid systems integrating multiple transfer learning models with feature selection methods, such as linear discriminant analysis combined with recurrent neural networks, have pushed boundaries further, yielding near-100% metrics in training and high validation scores. Such strategies reduce processing overhead while boosting generalization, positioning deep learning as a transformative force in accessible ophthalmic diagnostics [45][46].

## Unsupervised Learning for Pattern Discovery in Eye Diseases

Unsupervised learning is a machine learning approach where algorithms analyze and identify patterns in data without predefined labels or explicit guidance. Unlike supervised learning, which relies on labeled datasets to train models, unsupervised learning discovers hidden structures or relationships within unlabeled data. Common techniques include clustering (K-means, hierarchical clustering) to group similar data points and dimensionality reduction (principal component analysis) to simplify complex datasets while preserving key features. In the context of eye disease classification, such as in Liang et al. (2020) [47], unsupervised learning extracts radiomic features from optical coherence tomography images to identify distinct patient clusters with varying treatment outcomes for diabetic macular edema. Similarly, Tang et al. (2020) [48] used it for anomaly detection in corneal microscopy images, showing its ability to uncover novel biomarkers without prior knowledge of disease characteristics. This approach is particularly valuable in medical imaging, enabling the discovery of new patterns, improving diagnostic accuracy, and facilitating personalized treatment strategies for complex conditions [49][50].

## Conclusion

Literature shows a strict worldwide intention for development of ML models for aiding eye image diagnosis. As most studies have relied on supervised learning, the need for labeling image datasets by human supervision for data training shows extensive necessity of clinical specialists and machine learning specialists; while unsupervised methodologies can decrease the effort needed for manual labeling but needs validations. Some imaging modalities are also less studied as well as the fundus images, that warrant further studies in this era.

## Conflict of Interest

None.
